# Difunctionalization of Alkenes and Alkynes via Intermolecular Radical and Nucleophilic Additions

**DOI:** 10.3390/molecules26010105

**Published:** 2020-12-28

**Authors:** Hongjun Yao, Wenfei Hu, Wei Zhang

**Affiliations:** 1 College of Biological Science and Technology, Beijing Forestry University, 35 Qinghua East Road, Beijing 100083, China; hongjuny@bjfu.edu.cn; 2 Department of Chemistry, University of Massachusetts Boston, 100 Morrissey Boulevard, Boston, MA 02125, USA; wenfei.hu@umb.edu

**Keywords:** difunctionalization, radical, nucleophilic, alkene, alkyne, photoredox, single electron transfer, vicinal-difunctionalization, distal-difunctionalization, photoredox, electrochemical reaction

## Abstract

Popular and readily available alkenes and alkynes are good substrates for the preparation of functionalized molecules through radical and/or ionic addition reactions. Difunctionalization is a topic of current interest due to its high efficiency, substrate versatility, and operational simplicity. Presented in this article are radical addition followed by oxidation and nucleophilic addition reactions for difunctionalization of alkenes or alkynes. The difunctionalization could be accomplished through 1,2-addition (vicinal) and 1,n-addition (distal or remote) if H-atom or group-transfer is involved in the reaction process. A wide range of moieties, such as alkyl (R), perfluoroalkyl (R_f_), aryl (Ar), hydroxy (OH), alkoxy (OR), acetatic (O_2_CR), halogenic (X), amino (NR_2_), azido (N_3_), cyano (CN), as well as sulfur- and phosphorous-containing groups can be incorporated through the difunctionalization reactions. Radicals generated from peroxides or single electron transfer (SET) agents, under photoredox or electrochemical reactions are employed for the reactions.

## 1. Introduction

Radical-based reactions such as homolytic bond cleavage, single electron transfer, atom or group transfer, radical addition, and radical coupling processes are important organic transformations. In addition to the success of radical polymerization for making synthetic polymers, organic radical reactions have also become an increasing important tools for preparation of small molecules through radical addition, cyclization, cascade bond formation, H-atom and group-transfer, and radical-radical coupling reactions [1,2]. Active researches on direct C-H bond functionalization [3], remote (distal) functionalization [4], Smile-type *ipso*-group rearrangement [5], cascade reaction [6], photoredox catalysis [7], and electrochemical reaction [8] have significantly fueled the chemistry of synthetic radicals.

**Scheme 1:**
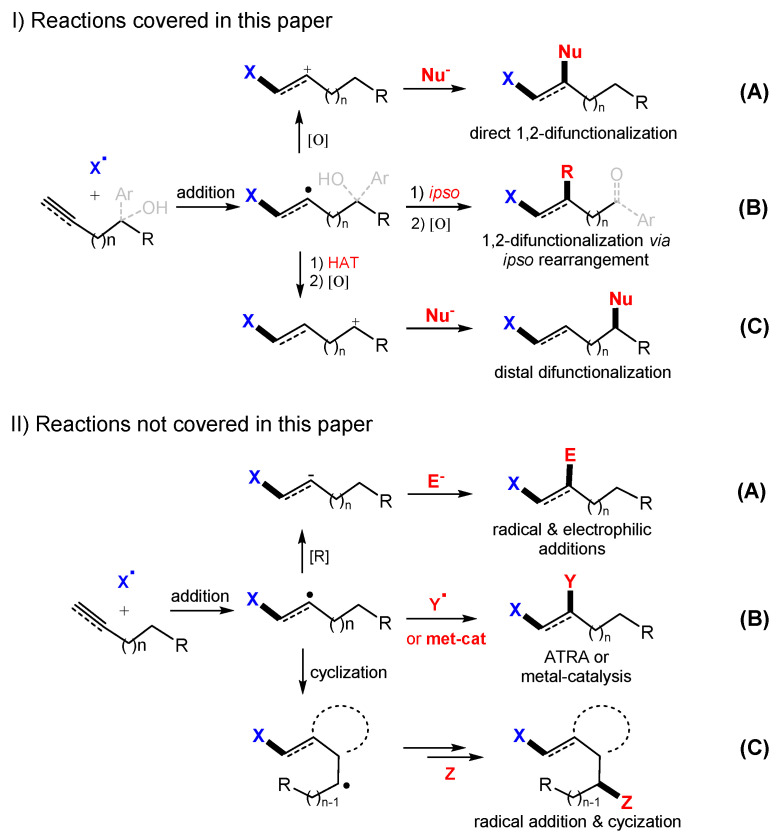
Radical-initiated difunctionalization reactions. (**I-A**), direct 1,2-difuctionalization. (**I-B**), 1,2-difuctionalization involving *ipso*-reaction process. (**I-C**), distal difunctionalization. (**II-A**), 1,2-difuctionalization involving electrophilic addition. (**II-B**), atom-transfer addition with or without metal catalysis. (**II-C**), reaction involving cyclization.

Radical difunctionalization of unsaturated carbon-carbon bonds is a topic of current interest due to its high synthetic efficiency, substrate versatility, and operational simplicity [9,10,11,12,13,14,15,16,17]. Presented in this article are one-pot radical addition, oxidation and nucleophilic addition reactions to incorporate two functional groups to alkenes or alkynes. The difunctionalization could be accomplished through direct 1,2-addition (Scheme 1I-A), 1,2-functionalization involving Smiles-type *ispo* aryl group migration (Scheme 1I-B) [4,5], or 1,n-functionalization involving H-atom transfer (Scheme 1I-C) [4,5,6]. The majority examples in this paper are direct 1,2-additions. Only a few cases are related to 1,2-functionalization through Smiles-type rearrangement and distal difunctionalizations through 1,5-H-atom transfer. A wide range of moieties, such as alkyl (R), perfluoroalkyl (R_f_), aryl (Ar), hydroxy (OH), alkoxy (OR), acetatic (O_2_CRRCO_2_), halogenic (X), amino (NR^2^), azido (N_3_), cyano (CN), as well as sulfur- and phosphorous-containing groups can be incorporated through difunctionalization reactions. Radical reactions initiated with peroxides (such as dicumyl peroxide (DCP), di-*t*-butyl peroxide (DTBP), and *t*-butyl hydroperoxide (TBHP)) [18], promoted by single electron transfer agents (such as hypervalent iodine reagents (HIRs), ceric ammonium nitrate (CAN), Mn(OAc)_2_, and Na_2_S_2_O_5_), under photoredox catalysis (such as Ru(bpy)_3_Cl_2_ and Ir(ppy)_3_) [7], and by electrochemical reactions [8] are included. In the radical-initiated difunctionalization reactions, the catalysts are critical for oxidative SET to convert the intermediate radicals to carbocations for the sequential reaction with nucleophiles.

This paper covers difunctionalization reactions which are initiated with a radical addition and ended with a nucleophilic addition. It is organized based on the nature of initial radicals including C-, N-, O-, P-, S-centered and perfluoroalkyl (R_f_) radicals. Reactions involving electrophilic addition (Scheme 1II-A), radical-radical coupling such as atom transfer radical addition (ATRA) [19] or metal-catalyzed coupling (Scheme 1II-B) [20], radical cyclization for ring formation (Scheme 1II-C) [21], and cascade reactions [6] involving more than two steps of radical additions are not included in this paper. Those type of reactions might be the topics of our future review articles.

## 2. Carbon Radical-Initiated Reactions

Presented in this section are carbon radical-initiated reactions and C-, O-, and N-containing moieties are served as the nucleophiles for the difunctionalizations. The initial carbon radicals could be generated by metal-containing SET agents, using peroxides, or under photoredox conditions. Most alkenes used for difunctionalization are styrene derivatives. Because addition of initial radicals to styrenes to form stabilized benzylic radicals is regioselective which could be readily oxidized to benzylic cations for nucleophilic additions.

**Scheme 2:**
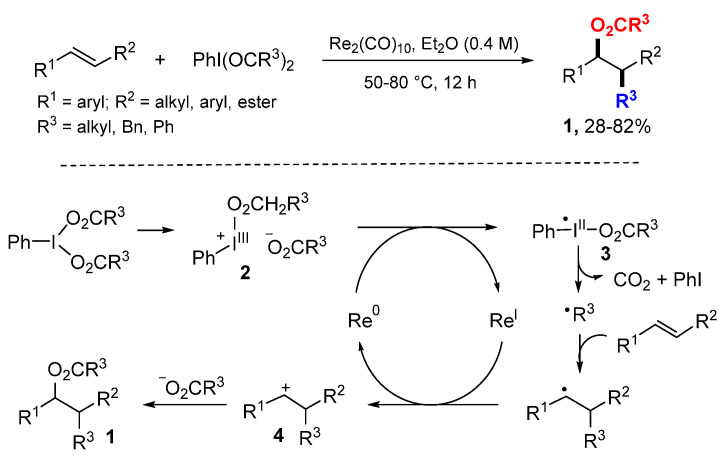
Re-catalyzed difunctionalization with HIRs.

The Du and Wang labs developed a highly efficient Re-catalyzed oxyalkylation of alkenes using hypervalent iodine reagents (HIRs) as a dual functionalization agent to provide alkyl radicals and carboxylic anions for additions (Scheme 2) [22]. In the reaction process, cationic species **2** generated through a heterolytic cleavage of an I-O bond from HIR are reduced by Re^I^ to afford radical iodine intermediates **3**. Homolytic breaking of the remaining I-O bond gives acyloxy radicals which undergo decarboxylation to generate alkyl radicals. Addition of alkyl radicals to alkene followed by oxidation with Re^I^ give cations **4**. Finally, nucleophilic attack of **4** by carboxylic anions gives difunctionalized products **1**.

**Scheme 3:**
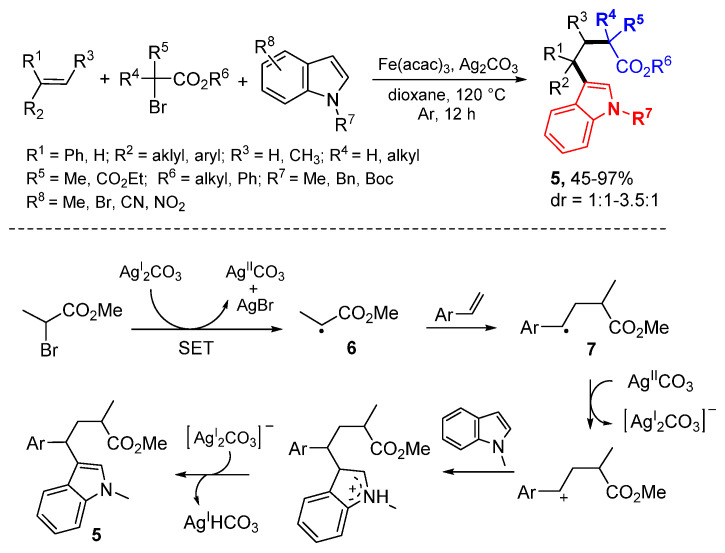
Ag-catalyzed 1,2-alkylarylation of styrenes.

The Li group reported Ag-mediated 1,2-alkylarylation of styrenes with α-carbonyl alkyl bromides and indoles (Scheme 3) [23]. The Fe-salt is used as Lewis acid to stabilize the radicals.The initial alkyl radicals **6** produced from α-carbonyl alkyl bromides in the presence of 2 equiv of Ag^I^ salt add to styrenes to afford radicals **6** which are oxidized by Ag^II^ to cations and then undergo the Friedel–Crafts reaction with indoles to give products **5**.

**Scheme 4:**
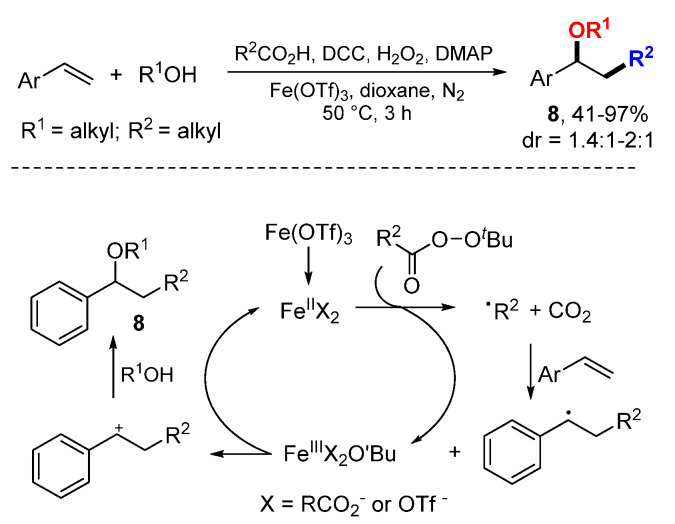
Fe-catalyzed alkyl etherification with aliphatic acids.

The Bao group reported Fe-catalyzed decarboxylative alkyl etherification of vinylarenes with aliphatic acids and alcohols (Scheme 4) [24]. Primary, secondary and tertiary aliphatic acids could be used as alkylating reagents, and primary and secondary alcohols as nucleophiles. Alkyl radicals generated from the reaction of Fe^II^X_2_ complex with peresters add to styrenes to form benzylic radicals which are oxidized to carbocations by Fe^III^ species and then undergo nucleophilic reaction to give products **8**.

**Scheme 5:**
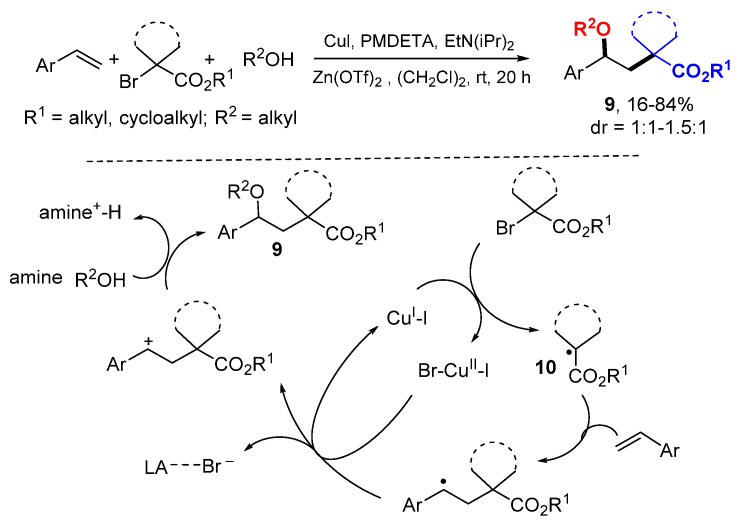
Cu-catalyzed difunctionalization of styrenes.

The Nishikat group reported a Cu-catalyzed reaction for difunctionalizing styrenes using α-bromocarbonyl compounds as a source for tertiary alkyl radicals and alcohols as nucleophiles (Scheme 5) [25]. Single electron transfer (SET) of Cu^I^ induces the formation of radicals **10** from α-bromocarbonyl compounds. The addition of radicals to styrenes followed by oxidation with Cu^II^ to benzylic cations and trapping with alcohol nucleophiles give products **9**. It was found that Lewis acids (LA), such as Zn(OTf)_2_, could accelerate the nucleophilic addition of alcohols.

**Scheme 6:**
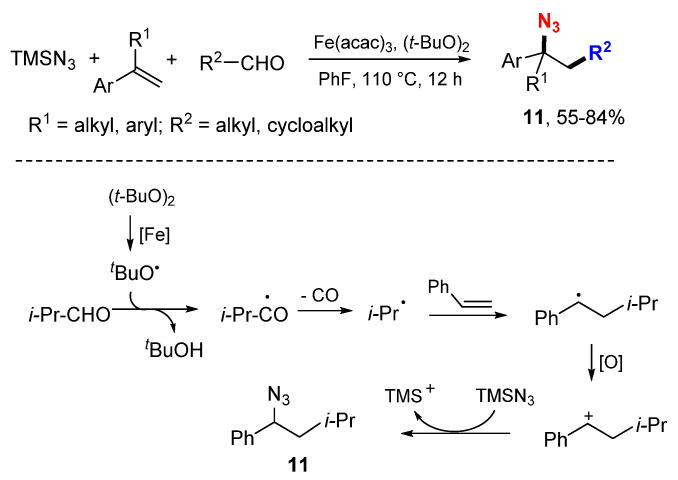
Fe-catalyzed alkylazidation of styrenes.

The Yang group reported Fe-catalyzed decarbonylative alkylazidation of styrenes with aliphatic aldehydes and trimethylsilyl azide (Scheme 6) [26]. Using (*t-*BuO)_2_ (DTBP) as an oxidant and radical initiator, a series of aliphatic aldehydes were converted to primary, secondary and tertiary alkyl radicals to react with styrenes followed by nucleophilic azidation with TMSN_3_. In the presence of the Fe catalyst, *t*-butoxy radical formed through the homolytic cleavage of DTBP abstracts the hydrogen from aldehydes followed by decarbonylation to give alkyl radicals which then react with styrenes to from benzylic radicals. Formation of cations by oxidization followed with the reaction of TMSN_3_ give products **11**. The authors also proposed another mechanism of radical coupling in the formation of products.

**Scheme 7:**
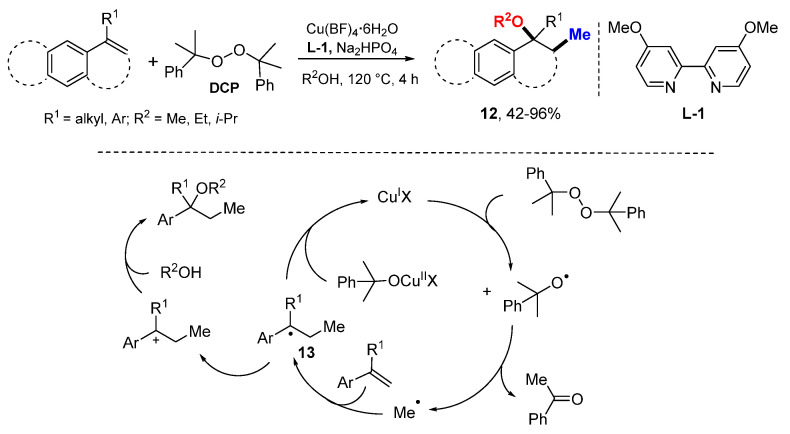
Cu-catalyzed methylalkoxyl difunctionalization of styrenes.

The Zhu group reported a Cu-catalyzed methylalkoxy difunctionalization of alkenes using dicumyl peroxide (DCP) as a methyl source and alcohols as nucleophiles (Scheme 7) [27]. The *t*-alkoxy radical generated from the cleavage of DCP undergoes β-scission to release the methyl radical. It is captured by alkenes to produce benzyl radicals **13** which are oxidized to carbeniums by Cu^II^ and then react with alcohols to furnish the methylated ether products **12**. 

**Scheme 8:**
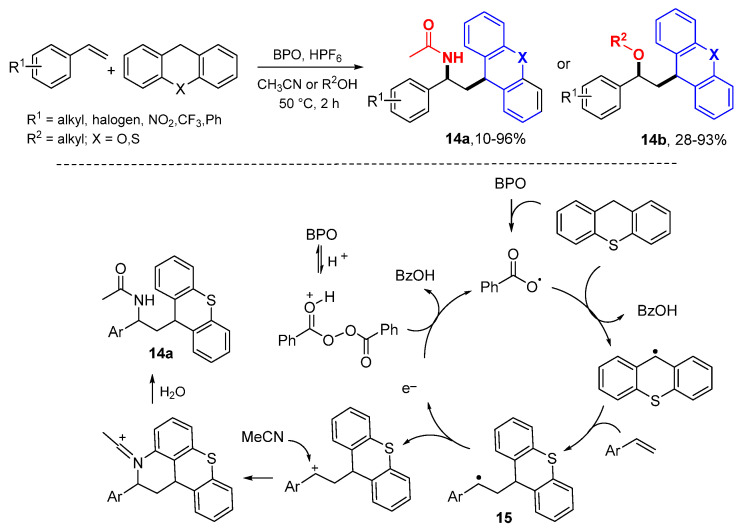
Nitro- and oxyalkylation of styrenes.

The Klussmann group reported a reaction for difunctionalization of styrenes with carbon radicals derived from thioxanthene/xanthene and thiophenols followed by nucleophilic addition of acetyl nitrile or alcohols (Scheme 8) [28]. The combination of benzoyl peroxide (BPO) with HPF6 is a key factor of this reaction. The benzoyloxyl radical generated from BPO abstracts H-atom from thioxanthene to give a new radical which then adds to styrenes to form the benzylic cations **15** after oxidation with BPO in the presence of HPF6. Acetonitrile or alcohols react with the carbocations in the fashion of a Ritter reaction to generate products **14a** or **14b**.

**Scheme 9:**
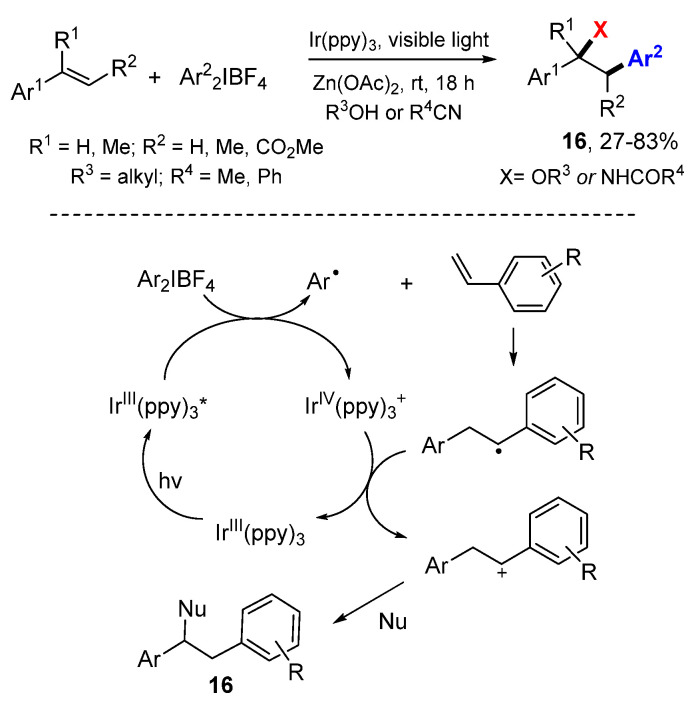
Aryl/alkyloxy or alkylamino difunctionalization of styrenes. * excited catalyst.

The Greaney group reported photoredox catalytic arylalkoxy or alkylamino difunctionalization of styrenes using Zn(OAc)_2_ to enhance the reaction process (Scheme 9) [29]. Aryl radicals generated from the reduction of Ar_2_IBF_4_ under the catalysis of Ir^III^ add to styrenes to form benzylic radicals which are oxidized with Ir^IV^ to cations. Addition of nucleophiles (alcohols, H_2_O or nitriles) afford difunctionalized products **16**. 

**Scheme 10:**
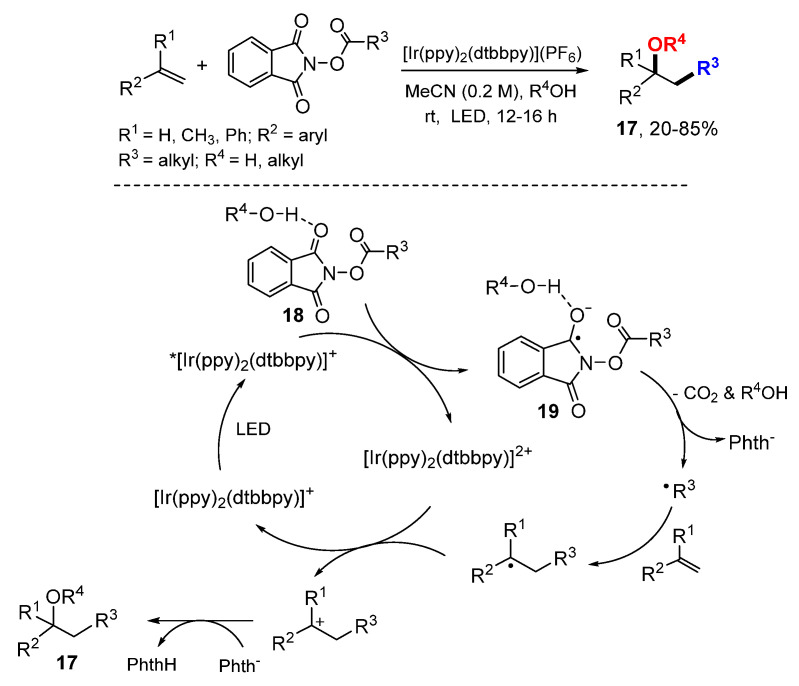
Visible-light-mediated alkyloxylation. * excited catalyst.

The Glorius lab developed a method for alkyloxylation of styrenes through photo electron transfer-initiated reaction of *N*-(acyloxy)phthalimides (Scheme 10) [30]. Water and alcohols act as nucleophiles and hydrogen-bond donors to afford alcohol and ether products **17**. The reactions involve SET of light-emitting diode (LED) excited [Ir(ppy)_2_(dtbbpy)]^+^ with the hydrogen bond complexes **18** to give radical anions **19**, which then undergo N-O bond cleavage followed by decarboxylation to afford alkyl radicals. Radical addition to olefin followed by oxidation with [Ir(ppy)_2_(dtbbpy)]^2+^ afford cations which are trapped by nucleophiles to afford alkyloxylated products **17**.

**Scheme 11:**
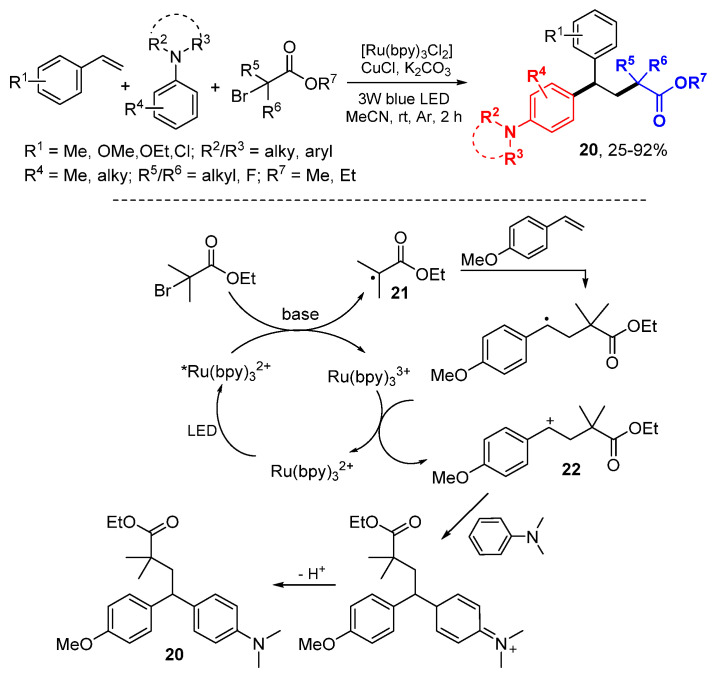
Alkyl & aryl difunctionalization of styrenes. * excited catalyst.

The Li lab reported an alkylaryl reaction of styrenes with α-carbonyl alkyl bromides and *N,N*-disubstituted anilines under photoredox catalysis of Ru(bpy)_3_Cl_2_ in the presence of a Cu salt (Scheme 11) [31]. The light excited catalyst Ru(bpy)_3_^2+^ transfers single electron to ethyl 2-bromo-2-methylpropanoates to form alkyl radicals **21** which add to styrenes, oxidized by Ru(bpy)_3_^3+^ to cationic intermediates **22**, followed by the Friedel–Crafts reaction to give products **20**.

**Scheme 12:**
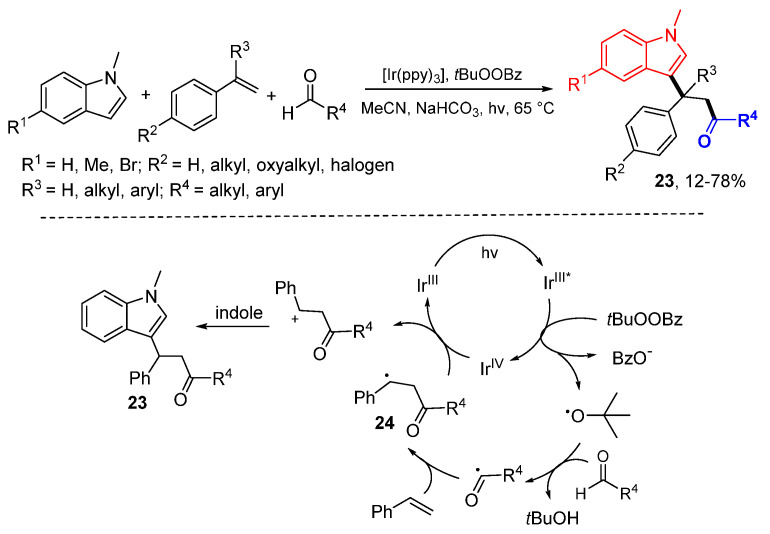
Acyl radical and *N*-alkylindole difunctionalization of styrenes. * excited catalyst.

The Klussmann group reported an Ir-promoted photoredox reaction of styrenes with aldehydes and *N*-alkylindoles (Scheme 12) [32]. Both aryl and aliphatic aldehydes could be used for the generation of acyl radicals. *t*-Butoxyl radical generated through the SET of photo-excited Ir^III^ and *t*BuOOBz (TBPB) react with aldehydes to form acyl radicals which add to styrenes to from **24** which then oxidized with Ir^IV^ to carbocations. The Friedel–Crafts reaction of carbocations with *N*-alkylindoles give final products **23**.

## 3. Nitrogen Radical-Initiated Reactions 

Presented in this section are azido radical (N_3_**·**)-initiated difunctionalization reactions. The azido radical can be generated from the Zhdankin’s reagent or TMSN_3_ [33].

**Scheme 13:**
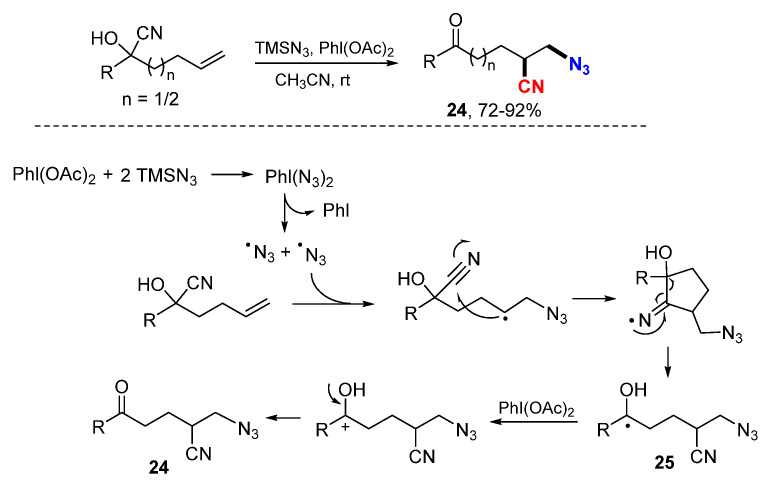
Azidocyanation of olefins involving cyano migration.

**Scheme 14:**
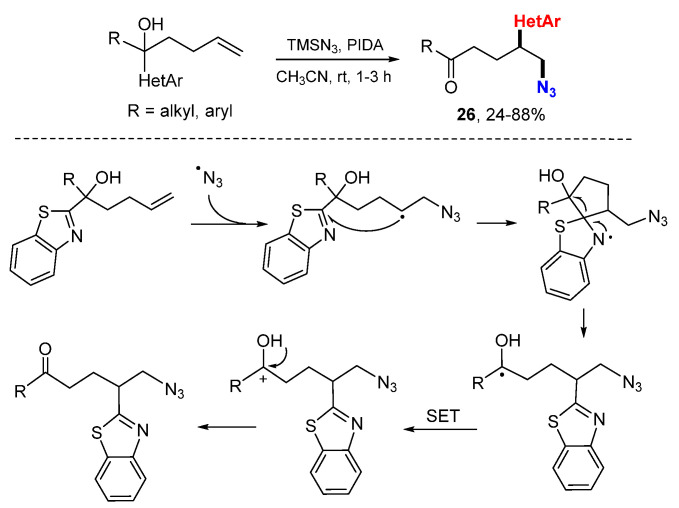
Azidoheteroarylation involving heteroaryl migration.

The Zhu group developed a transition-metal-free reaction for azidocyanation of alkenes involving 1,4- or 1,5-cyano migration (Scheme 13) [34]. An azido radical generated from the reaction of TMSN_3_ with PhI(OAc)_2_ adds to cyanohydrin alkenes. The resulted radicals undergo 1,4-cyano group migration (n = 1) through a cyclization and ring-opening process to give more stable hydroxyalkyl radicals **25**. Oxidation of the radicals with PhI(OAc)_2_ (PIDA) followed by deprotonation affords the desired product. The Zhu group applied this strategy for the reaction of γ-hydroxyalkenes in the synthesis of 1,2-difuctionalized compounds **26** involving the 1,4-heteroaryl group migration (Scheme 14) [35].

**Scheme 15:**
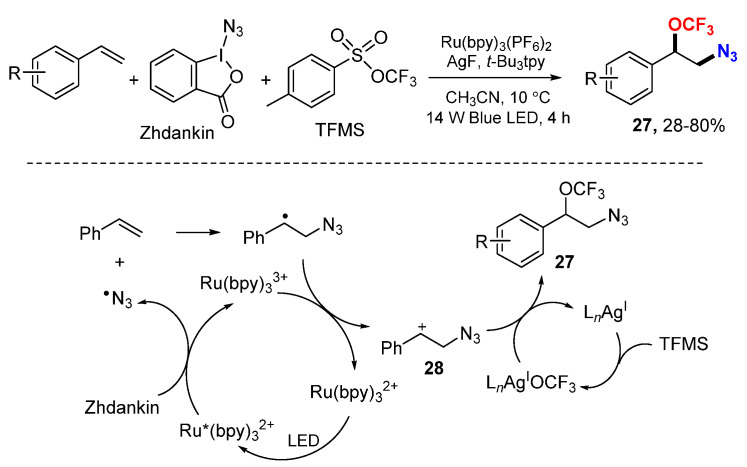
Ag/Ru-catalyzed azidotrifluoromethoxylation of styrenes. * excited catalyst.

The Tang group reported an azidotrifluoromethoxylation of styrenes with Zhdankin’s reagent and trifluoromethyl arylsulfonate (TFMS) (Scheme 15) [36]. The reactions were conducted under photoredox conditions using Ru and Ag as dual catalysts. [Ru(bpy)_3_]^2+^-Promoted formation of an azido radical from Zhdankin’s reagent adds to styrenes followed by oxidation to benzyl carbocations **28** and Ag-catalyzed trifluoromethoxylation give desired products **27**. The reaction tolerates a wide range of functional groups and is applicable to late-stage azidotrifluoromethoxylation of more complicated molecules. 

**Scheme 16:**
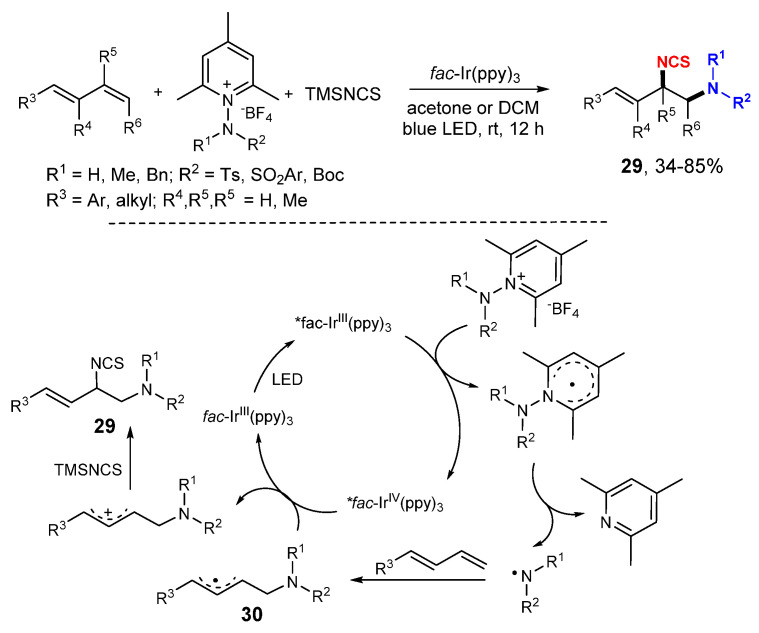
Difunctionalization of 1,3-dienes. * excited catalyst.

Zhu and coworkers developed a photoredox reaction for 1,2-difuctionalization of 1,3-dienes with *N*-aminopyridinium salts and TMSNCS (Scheme 16) [37]. Under the photocatalysis of *fac*-Ir(ppy)_3_, amino radicals derived from the *N*-aminopyridinium salts add to 1,3-dienes to form allylic radicals **30** which are oxidized to cations followed by a reaction with TMSNCS to give difunctionalized products **29**. 

## 4. Oxygen Radical-Initiated Reactions 

Difunctionalization reactions of unsaturated carbon double bonds initiated with the addition of oxygen radicals are limited in literature. The oxygen radicals described in this section are alkoxy, acyl and phthalimide *N*-oxyl (PINO) radicals which are generated from the homolytic cleavage of N-O or O-H bonds.

**Scheme 17:**
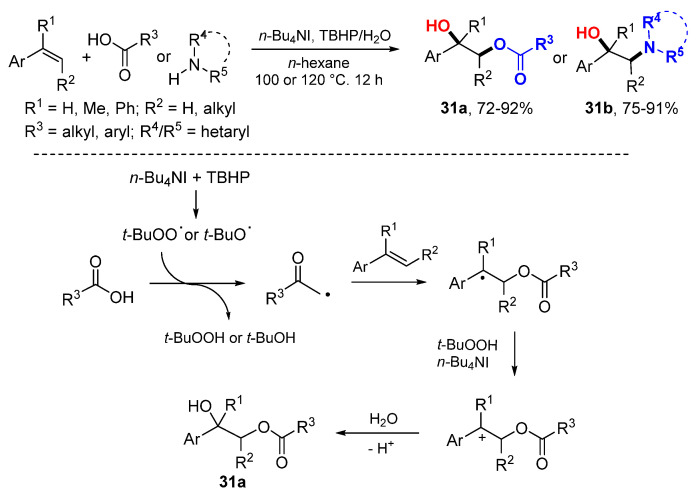
*n*-Bu_4_NI-catalyzed difuctionalization of alkenes.

The Zhu group developed *t*-butyl hydroperoxide (TBHP)-induced synthesis of *α*-hydroxy esters **31a** or *α*-hydroxy amines **31b** by the reaction of alkenes with carboxylic acids or amines in the presence of H_2_O and using TBHP (70% in water) as an oxidant (Scheme 17) [38]. *t*-Butoxyl and *t*-butylperoxy radicals generated from TBHP induce the formation of carboxyl radicals which add to styrenes followed by oxidation to benzyl cations and trapped with H_2_O to give products **31a**.

**Scheme 18:**
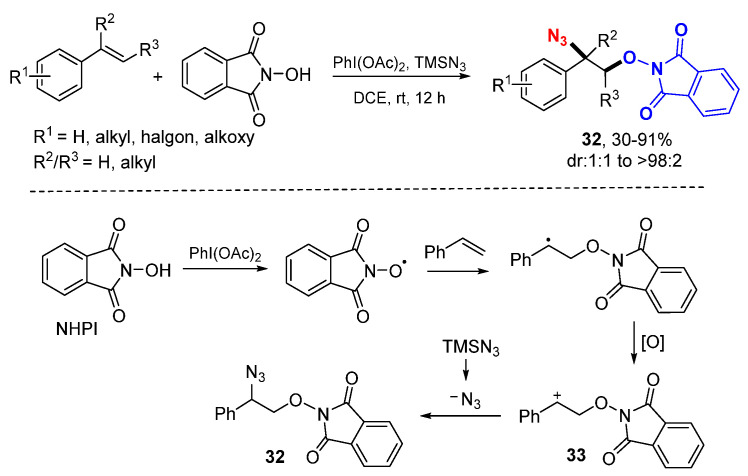
PINO radical-initiated difunctionalization of alkenes.

The Xia group reported a method for oxyazidation of alkenes with TMSN_3_ and *N*-hydroxyphthalimide (NHPI) for making 2-azido-2-(phenylethoxy)isoindolinone compounds **32** (Scheme 18) [39]. The oxygen-centered PINO radical generated from NHPI reacts with styrenes followed by oxidation to carbocations with PhI(OAc)_2_ to from cations **33** which react with N_3_^−^ to give oxyazidation products **32**.

**Scheme 19:**
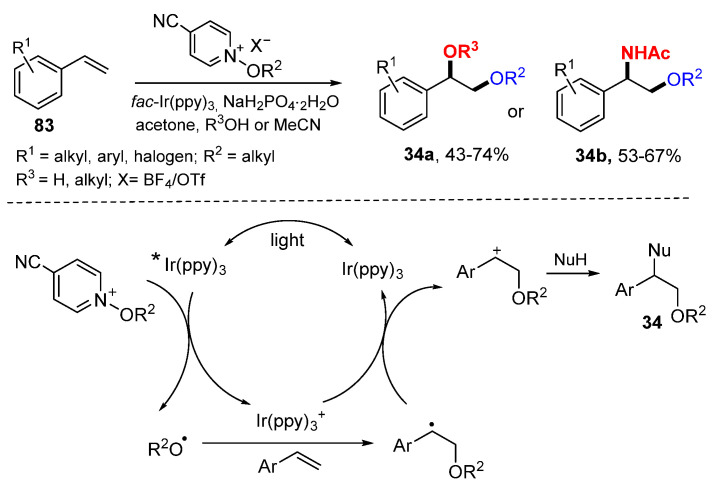
Dialkoxylation and aminoalkoxylation of alkenes. * excited catalyst.

The Dagousset group reported an alkoxy radical-initiated difunctionalization reaction of alkenes for the synthesis of functionalized diethers **34a** or amino ethers **34b** under batch or flow reaction conditions (Scheme 19) [40]. A radical/cationic addition mechanism was confirmed by electron paramagnetic resonance (EPR) studies. Alkoxy radicals generated from 4-cyano-substituted *N*-alkoxypyridinium salt under the catalysis of *fac*-Ir(ppy)_3_ add to alkenes followed by the SET of Ir(ppy)_3_^+^ to form corresponding carbocations. Nucleophilic addition of alcohols or acetonitrile gives difunctionalized products **34**.

## 5. Phosphorous Radical-Initiated Reactions 

Phosphorous radical-initiated difunctionalization reactions are highlighted in this section. So far we only found reactions of phosphinoyl [R_2_(O)P^·^] and phosphonyl [(RO)_2_(O)P^·^] radicals generated from corresponding phosphine oxides and phosphonates [41].

**Scheme 20:**
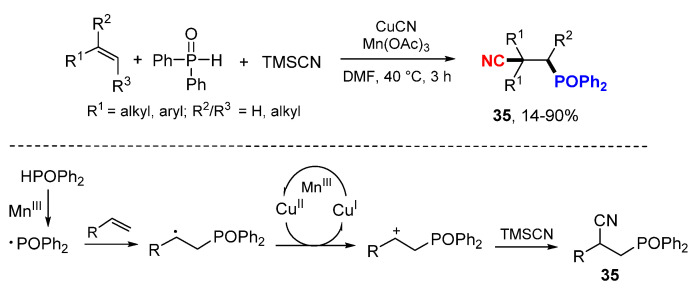
Phosphinoylation and cyanylation of alkenes.

The Zou and Zhang groups reported Cu-catalyzed difunctionalization of alkenes with diphenylphosphine oxide (HPOPh_2_) and trimethylsilylcyanide (TMSCN) (Scheme 20) [42]. The phosphinoyl radicals generated from the oxidation of HPOPh_2_ with Mn(OAc)_3_ react with alkenes which are oxidized with Cu^II^ and then cyanylated with CN^–^ to give products **35**.

**Scheme 21:**
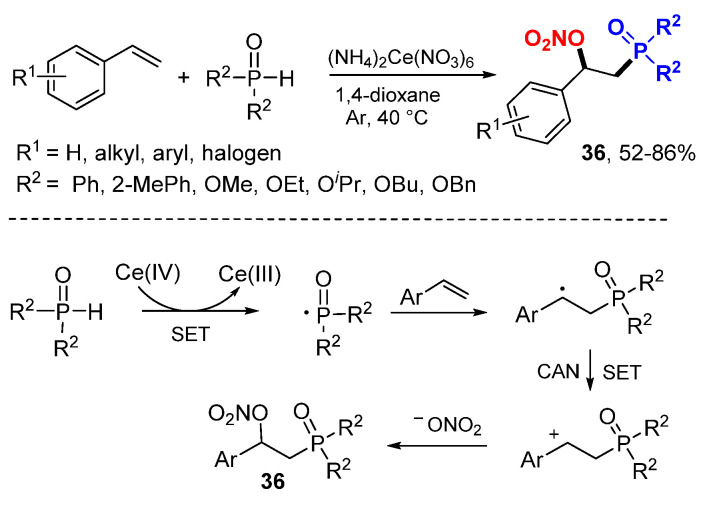
P-radical initiated difunctionalizations of styrenes.

Wang and coworkers reported Ce-catalyzed phosphinoylation and nitration reactions of alkenes (Scheme 21) [43]. CAN (NH_4_)_2_Ce(NO_3_)_6_was used as P-radical initiator and also nitrate donor to afford β-nitrooxyphosphonates **36**. The phosphinoyl radicals generated form phosphine oxides or phosphonates through a SET with Ce^IV^ add to alkenes to form benzylic cations after the second SET with CAN. Reaction of benzylic cations with nitrate give products **36**.

**Scheme 22:**
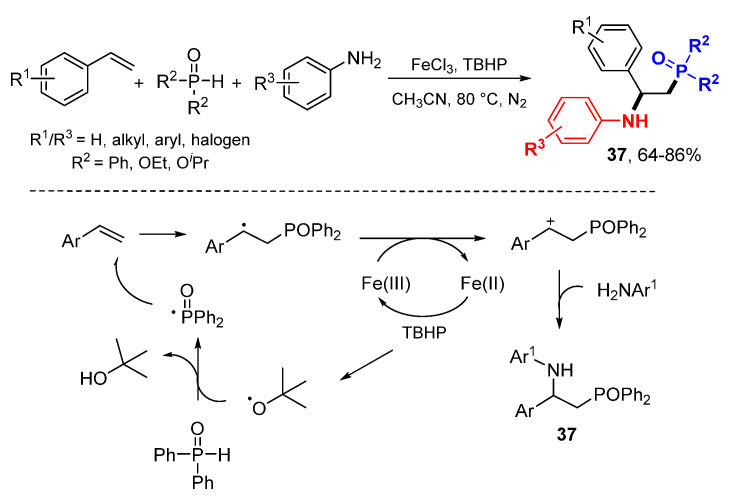
Phosphinoamination of styrenes.

Fang and coworkers reported TBHP and FeCl_3_-promoted difunctionalization of alkenes with phosphine oxides or phosphonates and anilines for the synthesis of products **37** (Scheme 22) [44]. In the reaction with diphenylphosphine oxide, phosphinoyl radicals add to alkenes to form carbocations after oxidation with FeCl_3_. Trapping of carbocations by amines afford corresponding α,β-phosphinoamination products **37**.

**Scheme 23:**
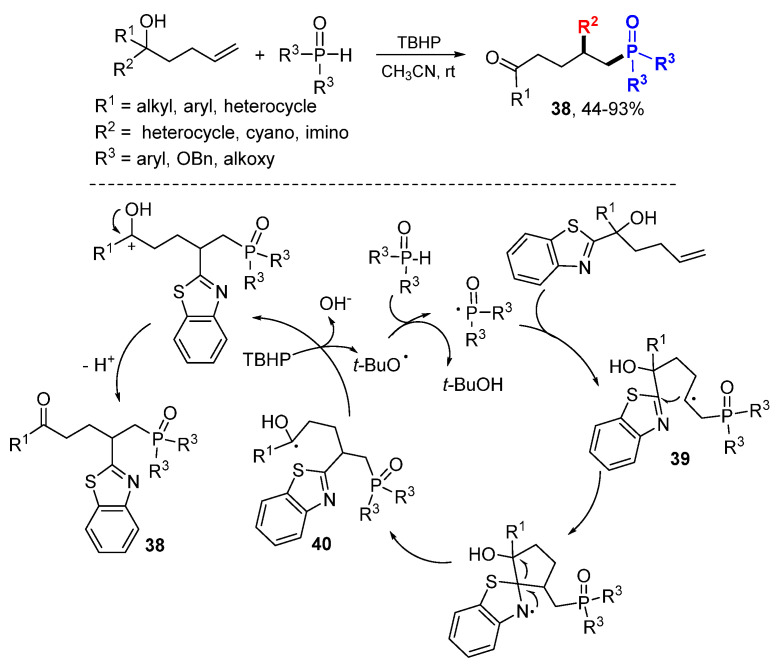
Radical-mediated phosphinoyl-functionalization of alkenes.

Using a similar strategy of heteroaryl group migration described in Scheme 14, the Zhu group accomplished phosphinoyl or phosphonyl radical-initiated difunctionalization of γ-hydroxyalkenes (Scheme 23) [45]. In the presence of TBHP, the P-radicals generated from phosphine oxides or phosphonates add to alkenes followed by 1,4-heteroaryl migration of **39** to form radicals **40**, oxidation of the radicals to cations to give products **38** after proton transfer. In addition to heteroaryl, other groups such as cyano and imino, could also be used as R^2^ for the migration.

## 6. Sulfur Radical-Initiated Reactions 

Sulfur-centered radicals, such as sulfenyl (RS^·^), sulfonyl [RS(O)_2_S^·^], and thiocyanate (SCN) radicals, have been used for difunctionalization reactions [41]. The sulfur radicals could be generated from corresponding reagents through homolytic N-S bond cleavage, single electron oxidation of KSCN, and electrochemical and photoredox catalytic reactions.

**Scheme 24:**
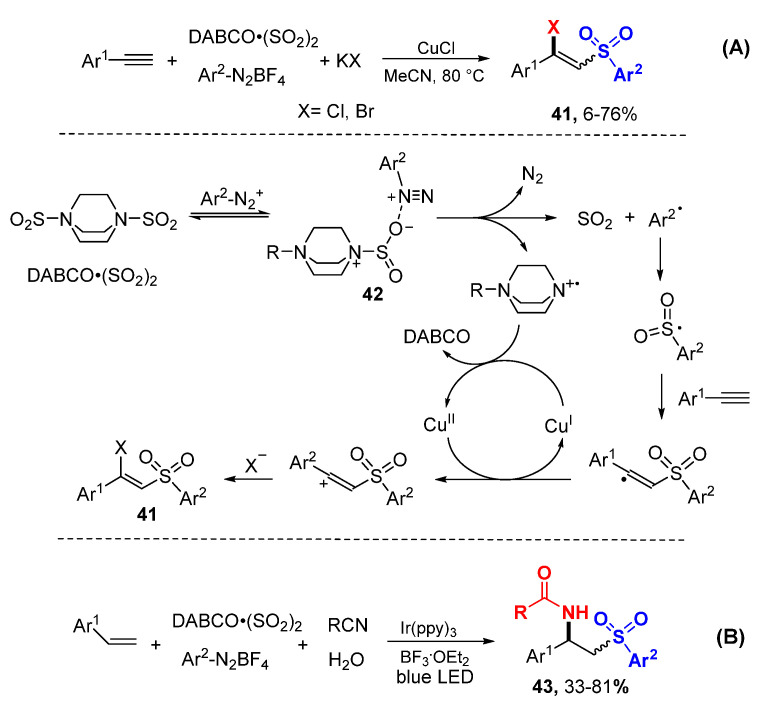
Halosulfonylation of aryl alkynes. (**A**) reaction of alkenes. (**B**) reaction of alkynes.

The Wu group reported a method for the synthesis of β-halo vinylsulfones through Cu-catalyzed reaction of alkynes with aryldiazonium tetrafluoroborates and DABCO·(SO_2_)_2_ (Scheme 24A) [46]. Arylsulfonyl radicals **42** generated from the reaction of aryldiazonium cations with DABCO·(SO_2_)_2_ attack alkynes to form stable alkenyl radicals which are oxidized to alkenyl cations with CuCl followed by nucleophilic addition with halides to give β-halo vinylsulfones **41**. The research group also extended the scope of the reaction of aryl alkenes under photoredox conditions and using alkyl nitriles as nucleophiles to afford aminosulfonylation products **43** (Scheme 24B) [47].

**Scheme 25:**
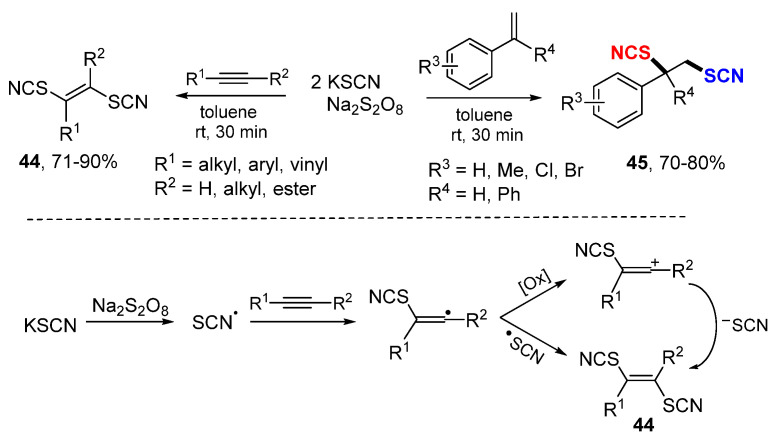
Dithiocyanation of alkynes and alkenes.

Majee and coworkers reported a method for dithiocyanation of alkynes and alkenes with KSCN and Na_2_S_2_O_8_ to give products **44** and **45**, respectively (Scheme 25) [48]. In the reaction of alkynes, the thiocyanate radical generated from the oxidation of KSCN adds to alkynes, followed by oxidization and reaction with KSCN to give dithiocyanation alkenes **44**. Other than nucleophilic addition of thiocyanate anion, coupling of thiocyanate radical is also a possible pathway.

**Scheme 26:**
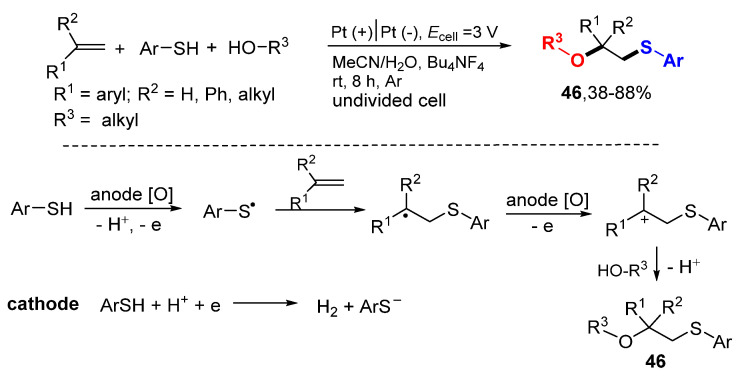
Electrochemical difunctionalization of styrenes.

The Han group developed an electrochemical oxysulfuration reaction of styrenes by addition of sufenylradicals and alkoxy nucleophiles (Scheme 26) [49]. The arylsufenyl radicals generated from thiophenol through the SET oxidation at the anode add to alkenes and then oxidize to carbocations at the anode. Trapping of the carbocations by alcohols affords the oxysulfuration products **46**.

**Scheme 27:**
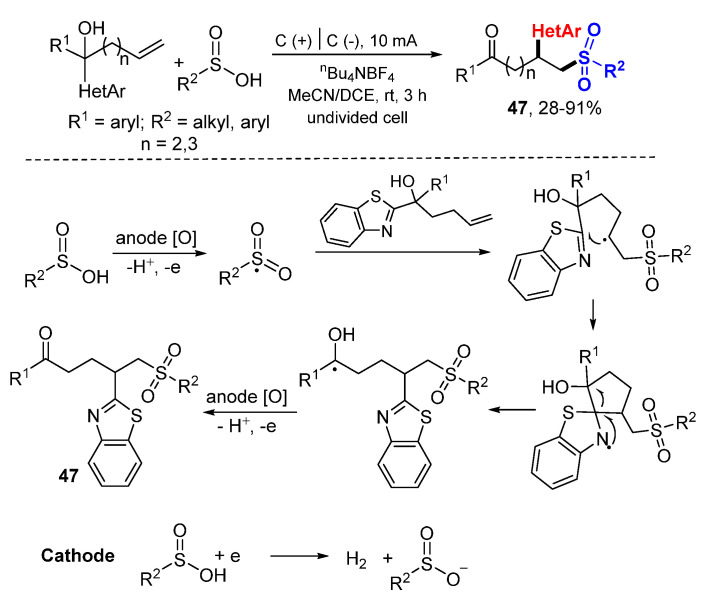
Electrochemical sulfonylheteroarylation of alkenes.

Guo and coworker reported electrochemical sulfonylheteroarylation reaction of alkenes involving heteroaryl group migration (Scheme 27) [50]. The arylsulfonyl radicals generated from sulfinic acid at the anode add to benzothiazole ring for heteroaryl migration followed by single-electron oxidation at the anode to afford cations and then products **47** after deprotonation. This *ipso*-aryl group migration process is similar to those presented in Scheme 14 and Scheme 23. 

**Scheme 28:**
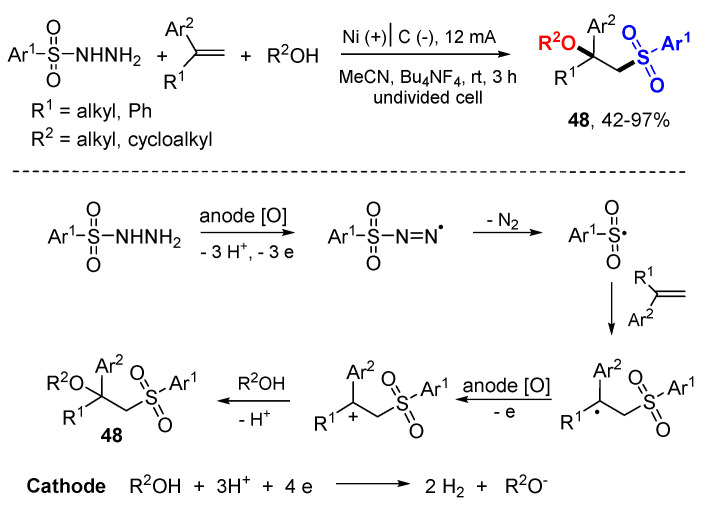
Electrochemical oxidative alkoxysulfonylation of alkenes.

An electrochemical oxidative alkoxysulfonylation of alkenes with sulfonyl hydrazines and alcohols have been reported by the Lei group (Scheme 28) [51]. The reactions were carried out at room temperature and only molecular nitrogen and hydrogen are released as byproducts. Sulfonyl radicals generated from sulfonyl hydrazines through oxidation at anode react with alkenes and then undergo second oxidation at anode to form benzyl cation intermediates. Nucleophilic attack of cations with alkoxyl anions give alkoxysulfonylation products **48**. The alkoxyl anions are generated from alcohols on the cathode.

**Scheme 29:**
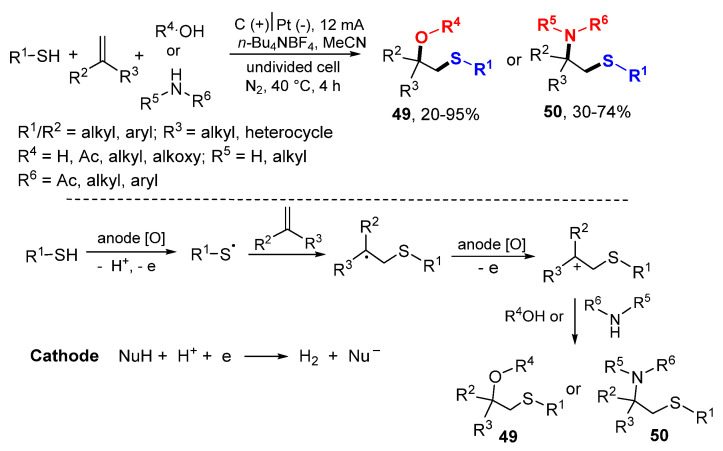
Electrochemical oxysulfenylation and aminosulfenylation of alkenes.

The Lei group also reported sulfenyl radical-initiated oxysulfenylation and aminosulfenylation of alkenes using thiophenols/thiols as thiolating agents and alcohols/amines as nucleophiles (Scheme 29) [52]. Under electrochemical reaction conditions, thiophenols/thiols are converted to sulfenyl radicals for addition to alkenes followed by anode oxidation of resulting radicals to cations and nucleophilic addition with alcohols or amines to give oxysulfenylation products **49** and aminosulfenylation products **50**, respectively.

**Scheme 30:**
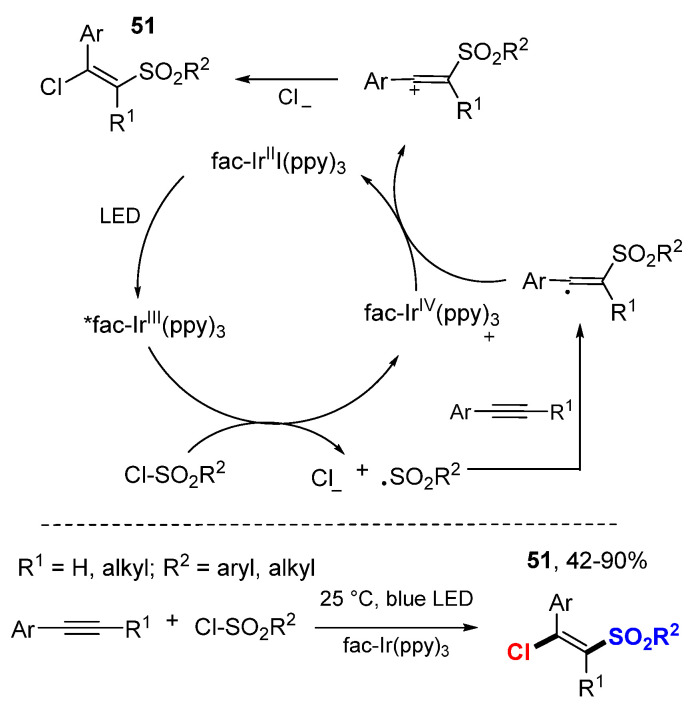
Chlorosulfonylation of arylalkynes. * excited catalyst.

Han and coworkers reported a photoredox-catalyzed chlorosulfonylation reaction of alkynes using sulfonyl chlorides as both sulfonyl radical and chlorine sources (Scheme 30) [53]. A variety of (*E*)-selective β-chlorovinyl sulfones **51** were prepared from terminal and internal alkynes.

**Scheme 31:**
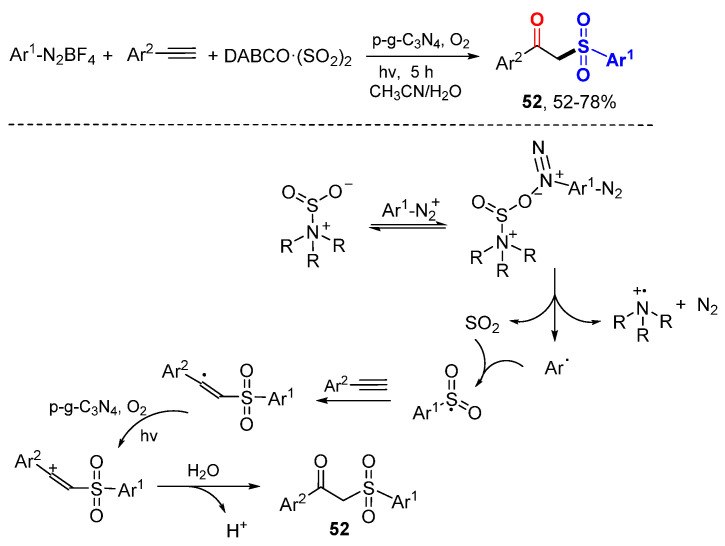
Light-promoted hydrosulfonylation of arylalkynes.

The Nie and Niu group reported the preparation of β-ketosulfones **52** via graphitic carbon nitride (p-g-C_3_N_4_)-photocatalyzed hydrosulfonylation of alkynes in aerobic aqueous medium (Scheme 31) [54]. The heterogeneous semiconductor is recyclable at least 6 times without significant reducing activity. Arylsulfonyl radicals generated from the complex of DABCO·(SO_2_)_2_and Ar^1^-N_2_BF_4_ add to arylalkynes to form arylalkenyl radicals and then arylalkenyl cations after SET oxidation. Hydrolysis of the cations give β-keto sulfones **52**.

**Scheme 32:**
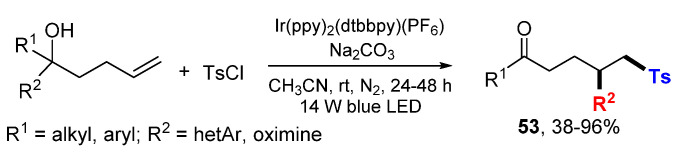
Heteroarylsulfonylation and oximinosulfonylation of alkenes.

The Zhu group reported photoredox heteroarylsulfonylation and oximinosulfonylation of unactivated alkenes via sulfonyl radical addition followed by heteroaryl or oximino group migration and oxidation of hydroxyl to ketone to afford products **53** (Scheme 32) [55]. The reaction process is similar to that presented in Scheme 14.

## 7. Fluorocarbon Radical-Initiated Reactions 

Fluorine atom and fluorinated alkyl groups could be used to improve molecules’ stability, bioavailability, and other physical, chemical and biological properties [56]. The development of new synthetic methods to introducing flouring-containing groups, such as CF_2_R, CF_3_ and multi-carbon perflourinated alkyls (R_f_), is a topic of current interest in both organic and medicinal chemistry [57]. Radical-initiated difunctionalization is a good approach for the synthesis of fluorinated compounds [58]. Most fluorocarbon radicals presented in this section are generated by metal-catalyzed photo-redox or electrochemical reactions from the substrates including Umemoto’s [59], Togni’s [60], and Langlois’ (CF_3_SO_2_Na) reagents. Perfluoroalkanesulfonyl chloride (CF_3_SO_2_Cl), α-bromo-2,2-difluoroacetates, bromodifluoromethylphosphonates, and perfluoroalkyl-containing hypervalent iodines are also good sources for fluorocarbon radicals.

**Scheme 33:**
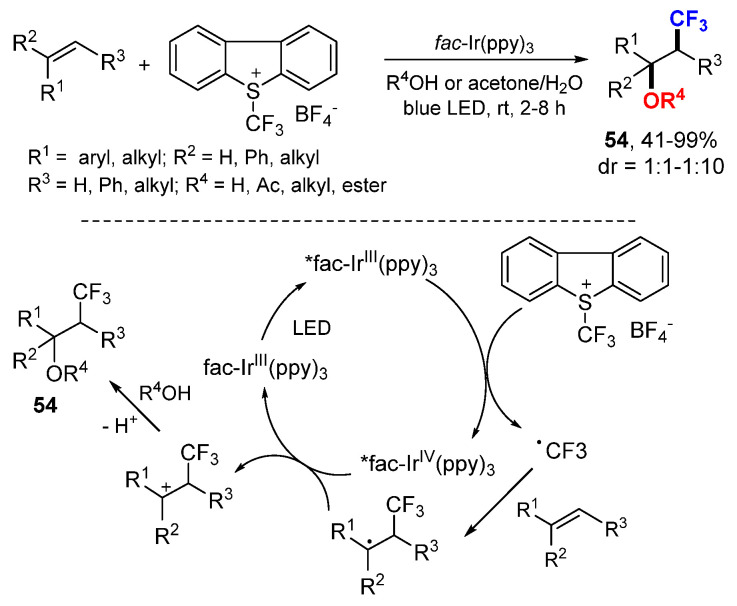
Photoredox catalyzed oxytrifluoromethylation of alkenes. * excited catalyst.

The Koike and Akita group developed a photoredox catalyzed oxytrifluoromethylation reaction of alkenes using Umemoto’s reagent as the CF_3_ source (Scheme 33) [61]. In this catalytic system, the sunlight is an efficient light source. The reaction process includes the generation of a CF_3_ radical from Umemoto’s reagent by photo catalytic SET, addition of CF_3_ radical to alkenes to form intermediate carbon radicals, oxidation to carbocation intermediates by catalytic SET, and alcohol nucleophilic addition give products **54**.

**Scheme 34:**
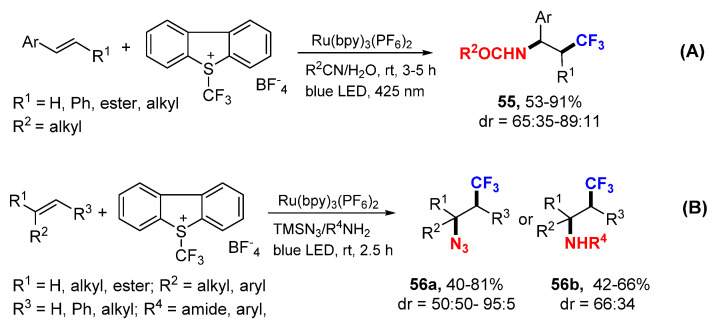
Difunctionalization using Umemoto’s reagent. (**A**) reaction with R_2_CN/H_2_O. (**B**) reaction with TMSN_3_/R_4_NH_2._

The Koike and Akita group also reported photoredox reaction of alkenes using Umemoto’s reagent as CF_3_ source and Ru(bpy)_3_(PF_6_)_2_ as a catalyst for the synthesis of aminotrifluoromethylation products **55** (Scheme 34A) [62]. The Magnier and Masson group used the similar reaction conditions in the synthesis of *β*-trifluoromethylated azides **56a** and amines **56b** (Scheme 34B) [63].

**Scheme 35:**
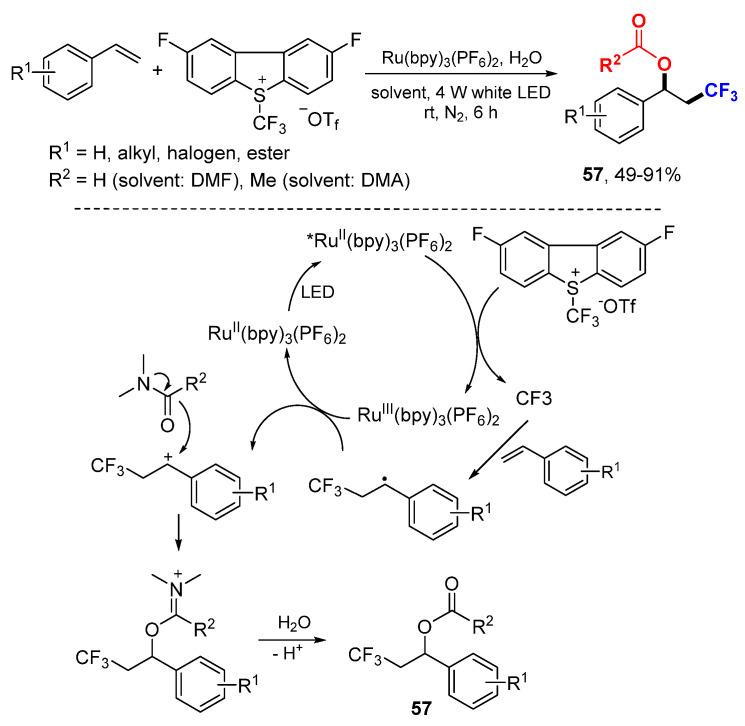
Acyloxytrifluoromethylation of styrenes. * excited catalyst.

The She and Li group reported acyloxytrifluoromethylation of alkenes with the Umemoto II reagent using Ru(bpy)_3_(PF_6_)_2_ as a photoredox catalyst (Scheme 35) [64]. *N,N*-Dimethylformamide (DMF) was employed as a solvent and also an acetylation reagent. CF_3_ radical generated from the Umemoto’s reagent through catalytic SET adds to arylalkenes to give stable benzyl radicals which are oxidized to benzyl carbocations followed by nucleophilic addition of DMF to give imine intermediates and then hydrolyzed to give desired products **57**.

**Scheme 36:**
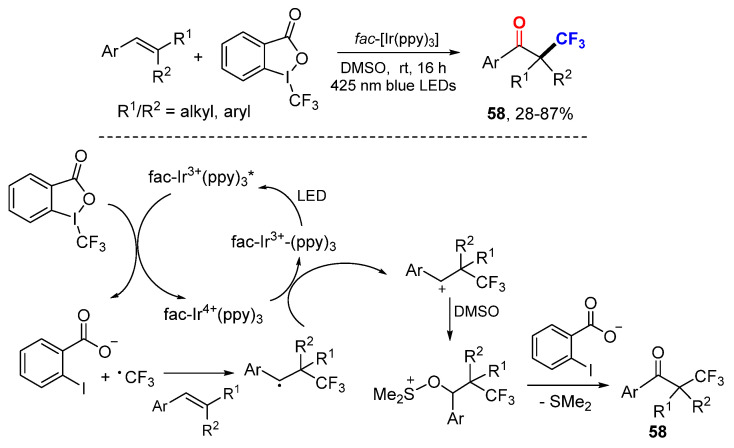
Photocatalytic keto-trifluoromethylation of styrenes. * excited catalyst.

The Koike and Akita group reported trifluoromethylation-initiated reaction for the synthesis of α-trifluoromethylated ketones **58** (Scheme 36) [65]. CF_3_ radical generated from the Togni’s reagent II through photocatalytic reductive SET of *fac*-[Ir^III^(ppy)_3_] adds to alkenes followed by oxidative SET gave β-CF_3_-substituted carbocationic intermediates. Nucleophilic attack of DMSO to carbocations affords alkoxysulfonium intermediates which then react with *o*-iodobenzoate for Korblum oxidation to give final products **58**.

**Scheme 37:**
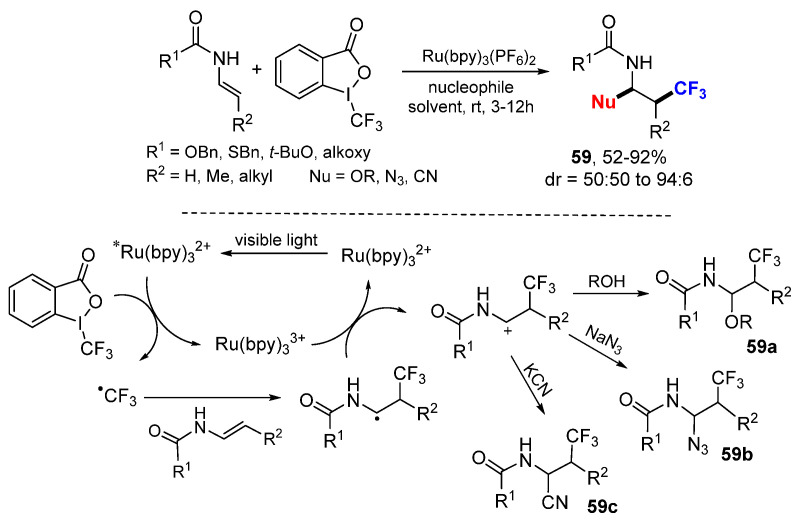
Difunctionalization of enecarbamates. * excited catalyst.

The Magnier and Masson group reported a photoredox-catalyzed carbotrifluoromethylation of enecarbamates using Togni’s reagent II as the CF_3_ source and various O-, N-, and C-containing compounds as nucleophiles (Scheme 37) [66]. The CF_3_ radical generated from Togni’s reagent by reductive photoredox SET adds to enecarbamates to form α-amido radicals which are quickly oxidized to acyliminium cations by a SET process. Nucleophilic trapping by alcohol, NaN_3_, or KCN affords the corresponding trifluoromethylated adducts **59a**–**c**.

**Scheme 38:**
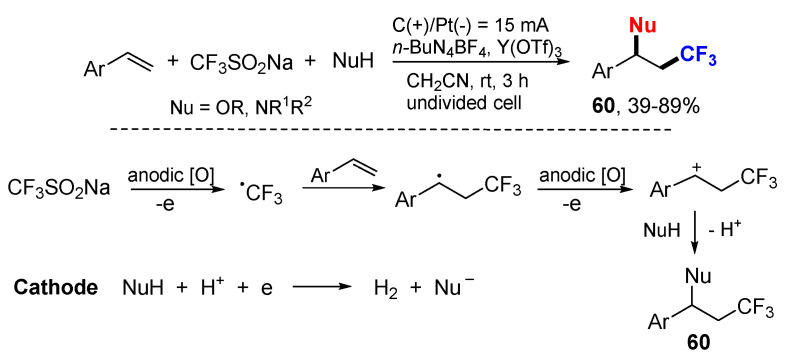
Trifluoromethylative Difunctionalization of Alkenes Using CF_3_SO_2_Na.

The Lei group reported an electrochemical reaction for oxytrifluoromethylation and aminotrifluoromethylation of alkenes using sodium trifluoromethanesulfinate (CF_3_SO_2_Na) as the source of CF_3_ radical (Scheme 38) [67]. CF_3_SO_2_Na is oxidized at the anode via SET to afford CF_3_ radical which adds to styrenes to generate benzylic radicals followed by oxidation to carbocations. Subsequent nucleophilic attack on the carbocations produces desired products **60**.

**Scheme 39:**
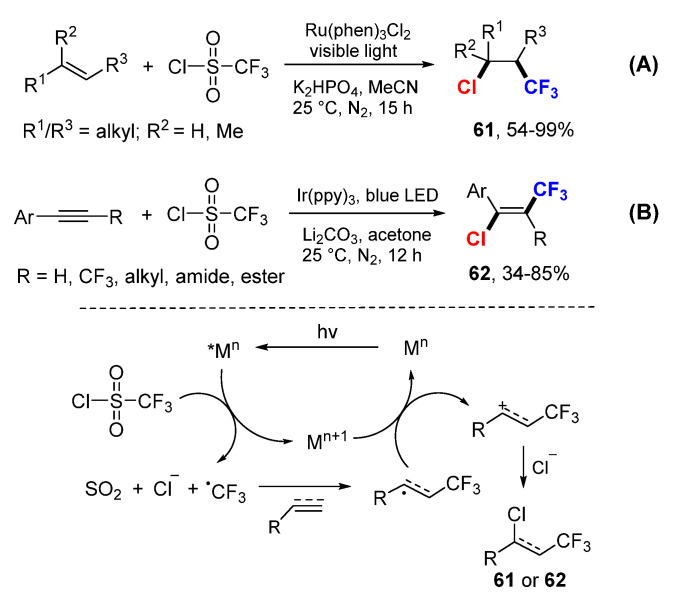
Chlorotrifluoromethylation of alkenes and alkynes. (**A**) Ru(phen)_3_Cl_2_-catalyzed reaction. (**B**) Ir(ppy)3-catalyzed reaction. * excited catalyst.

Han and coworkers reported a photoredox catalytic reaction for 1,2-chlorotrifluoromethylation of alkenes using CF_3_SO_2_Cl as a source for CF_3_ radical and chloride ion (Scheme 39A) [68]. The CF_3_ radical generated from CF_3_SO_2_Cl through reductive SET of Ru catalyst add to alkenes, followed by oxidative SET to carbocations and nucleophilic addition of chloride to give chlorotrifluoromethylated products **61**. The Han group extended the scope of the reaction for alkynes to make vicinal chlorotrifluoromethylated alkenes **62** using Ir-based photoredox catalyst (Scheme 39B) [69]. The resultant trifluoromethyl substituted vinyl chlorides can be used for Suzuki coupling to make 1,1-bis-arylalkenes.

**Scheme 40:**
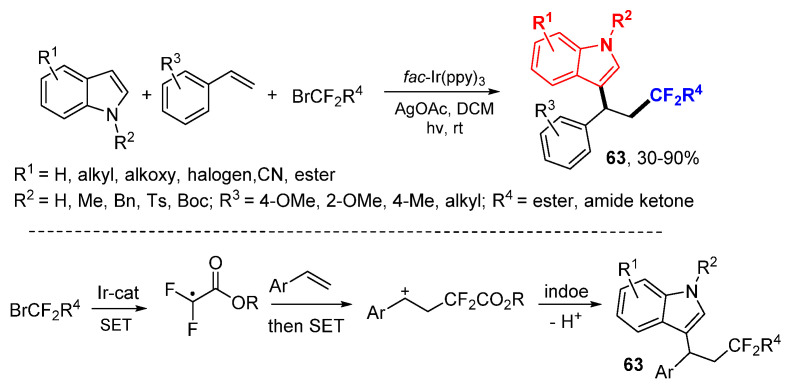
Photoredox difluoroalkylative difunctionalization of alkenes.

The Zhu group applied photoredox catalysis reaction for difluoroalkylarylation of styrenes using α-carbonyl difluoroalkyl bromides for CF_2_R radicals and indoles as nucleophiles in the synthesis of difluorolated indole derivatives **63** (Scheme 40) [70]. The CF_2_ R radicals generated from α-carbonyl difluoroalkyl bromides through reductive SET with Ir-photocatalyst add to alkenes followed by oxidative SET to cations and then react with indoles to give difluoroalkylated products **63** after base-mediated deprotonation.

**Scheme 41:**
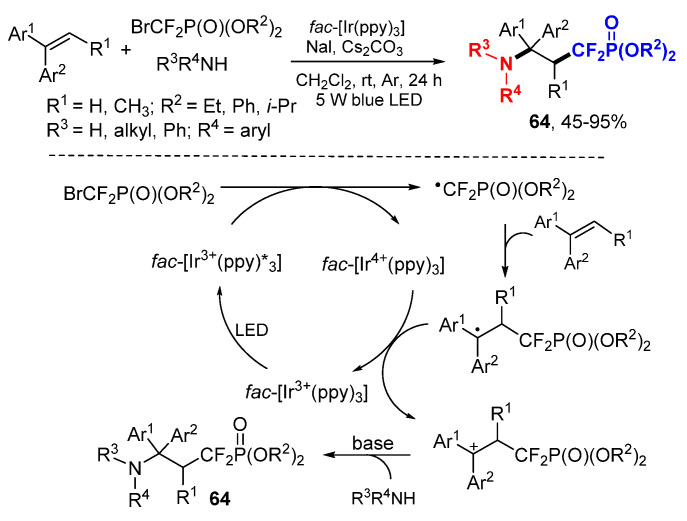
Photocatalyzed aminodifluoromethylation of alkenes. * excited catalyst.

Yang and coworkers reported a photocatalyzed reaction for aminodifluoromethylphosphonation of alkenes using bromodifluoromethylphosphonates as the source of CF_2_ radical (Scheme 41) [71]. The NaI was used as additive to stabilize the carbocation intermediates and increase the product yields. The difluorocarbon radicals produced through the SET process of bromodifluoromethylphosphonates add to alkenes followed by SET oxidation to cations for nucleophilic addition with amines to give the products **64** after deprotonation.

**Scheme 42:**
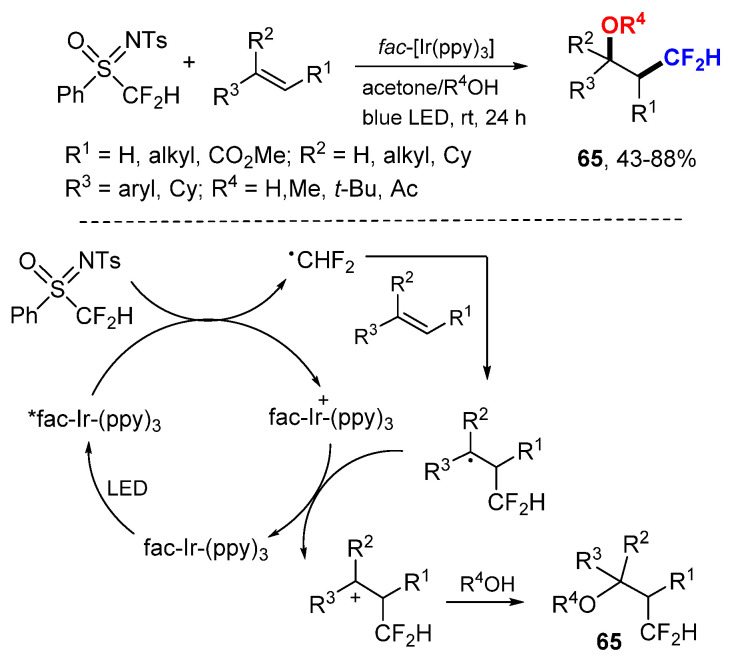
Photocatalytic oxydifluoromethylation. * excited catalyst.

The Koike and Akita group reported a photocatalytic oxydifluoromethylation of alkenes using shelf-stable and easy-to-handle *N*-tosyl-*S*-difluoromethyl-*S*-phenylsulfoximine as a CF_2_H source for the synthesis of β-CF_2_H-substituted products **65** including alcohols, ethers, and an ester (Scheme 42) [72]. The CF_2_H radical generated from *N*-tosyl-*S*-difluoromethyl-*S*-phenylsulfoximine by SET process reacts with alkenes to afford carbocationic intermediates after oxidative SET with Ir^(IV)^ and then solvolysis with ROH to produce oxydifluoromethylated products **65**.

**Scheme 43:**
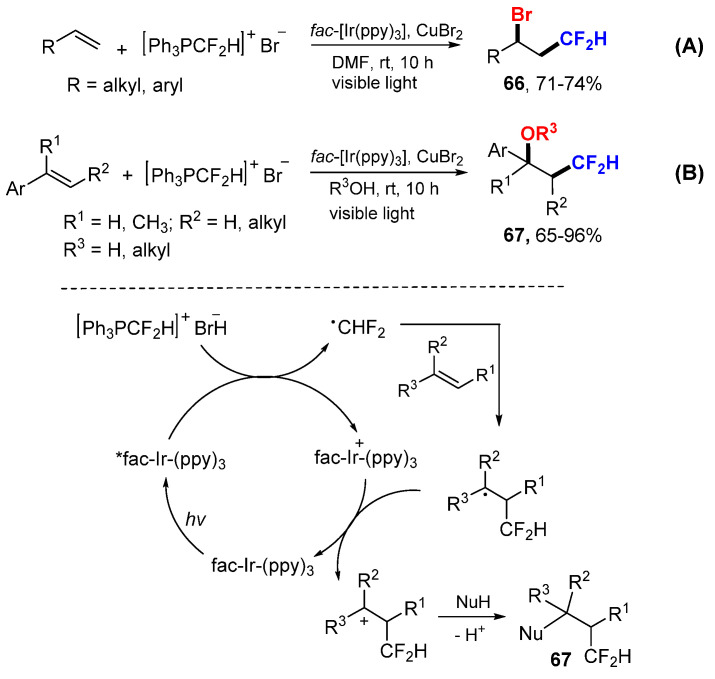
Bromodifluoromethylation and oxydifluoromethylation of alkenes. (**A**) reaction with Br^-^ as nucleophile. (**B**) reaction with R^3^OH as nucleophile. * excited catalyst.

The Qing group developed a photoredox-catalyzed bromodifluoromethylation reaction of alkenes using difluoromethyltriphenylphosphonium bromide for generating CF_2_H radical and also brominated anion as a nucleophile (Scheme 43A) [73]. The bromonated products **66** can be converted to CF_2_H-containing alkenes via an elimination process. The research group also reported a oxydifluoromethylation reaction of styrenes using difluoromethyltriphenylphosphonium bromide as a radical source and alcohols/water as nucleophiles for the synthesis of CF_2_H-containing alcohols and ethers **67** (Scheme 43B) [74].

**Scheme 44:**
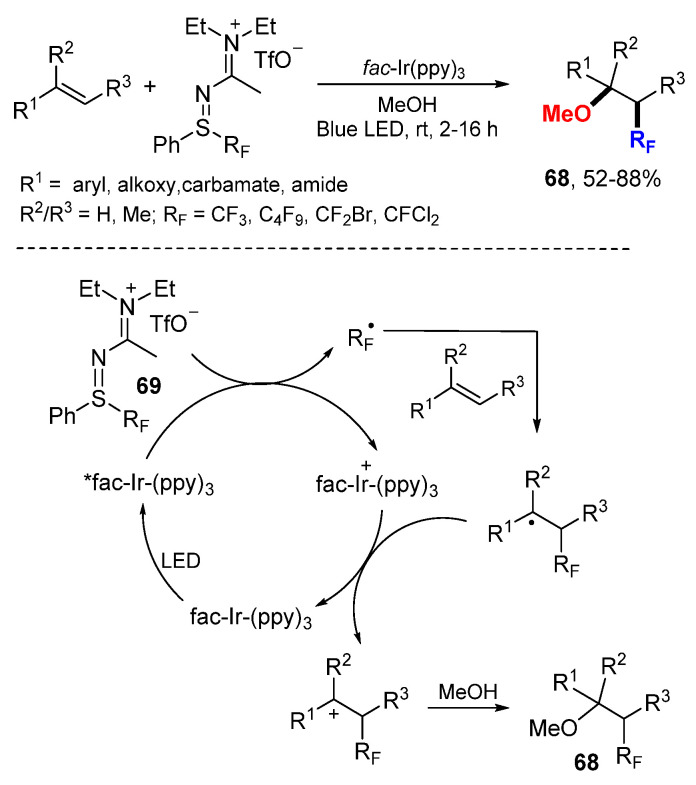
Oxyperfluoroalkylation with sulfilimino iminium ions. * excited catalyst.

The Magnier and Masson group employed S-perfluoroalkyl sulfilimino iminium ions **69** as a source of R_f_ radicals for oxyperfluoroalkylation of alkenes under photoredox catalysis conditions (Scheme 44) [75]. These stable perfluoroalkyl reagents containing CF_3_, C_4_F_9_, CF_2_Br, or CFCl_2_ can be readily prepared at gram scale from corresponding sulfoxides. The spin-trapping/electron paramagnetic resonance experiments confirmed that key radical intermediates involved in the radical/cationic process. R_f_ radicals generated from **69** by reductive SET add to alkenes to form stabilized benzylic radicals and then corresponding carbocations after oxidative SET. Final trapping with methanol provides corresponding products **68**.

**Scheme 45:**
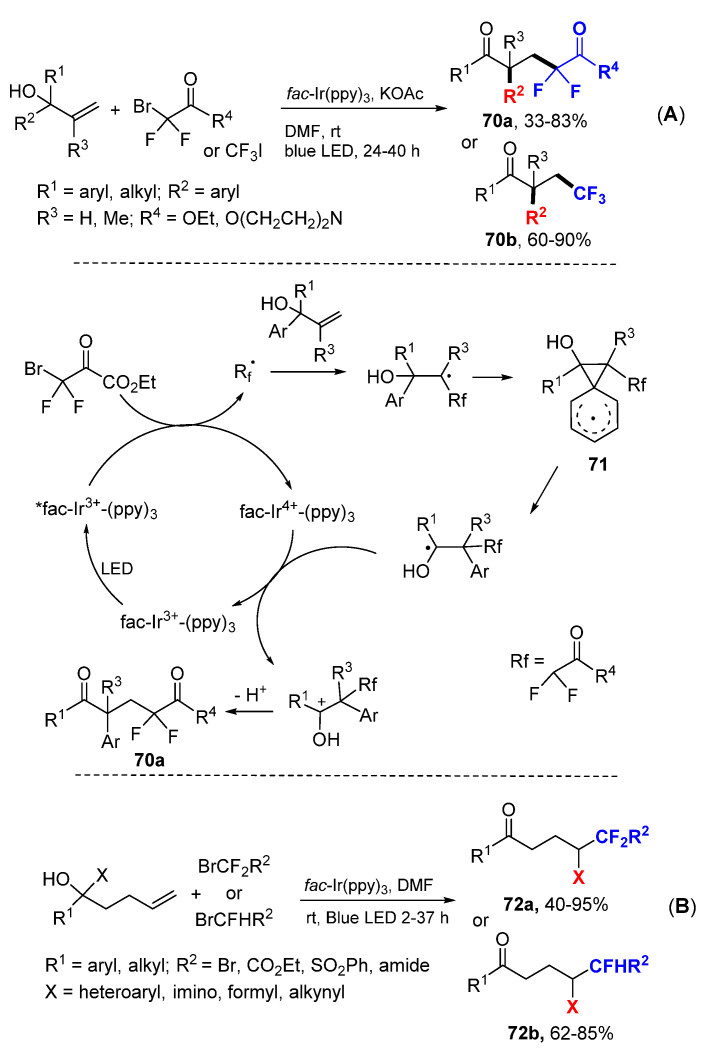
Difunctionalization involving a 1,2- or 1,4-group migration. (**A**) 1,2-migration reaction. (**B**) 1,4-migration reaction. * excited catalyst.

The Zhu group developed a visible light-promoted difluoroalkylarylation reaction of allylic alcohols for the synthesis of difluoro 1,5-dicarbonyl compounds **70** through radical addition and 1,2-aryl migration process (Scheme 45) [76]. The initial fluorocarbon radicals generated from ethyl 2-bromo-2,2-difluoroacetate or CF_3_I under SET add to allylic alcohols followed by *ipso* 1,2-aryl migration via spiro[2.5]octadienyl radicals **71** to give hydroxycarbon radicals. Oxidative SET of the radicals to corresponding carbocations followed by deprotonation give products **70**. The Zhu group extended the scope of the reaction for 1,4-aryl and other group migrations using *β*-hydroxy alkenes as starting materials for the synthesis of **72a** and **72b** [77].

**Scheme 46:**
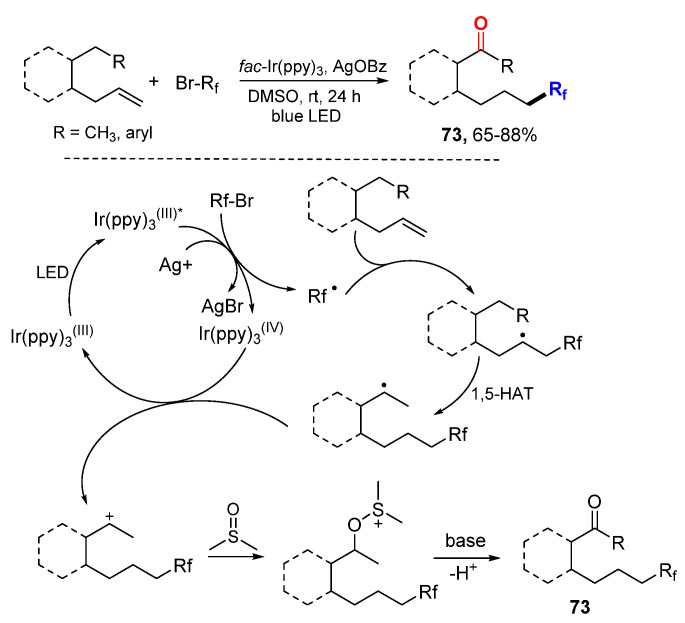
1,6-Oxyfluoroalkylation of alkenes. * excited catalyst.

The Ma and Li group reported a photoredox-catalyzed 1,6-oxyfluoroalkylation of alkenes using DMSO as an oxidant and also a solvent (Scheme 46) [78]. The perfluoroalkyl (R_f_) radicals are produced from R_f_Br through the photoredox of the Ir-catalyst in the assistance of the Ag salt. The benzylic radicals generated from the addition of R_f_ radicals to alkenes undergo 1,5-hydrogen atom transfer (1,5-HAT) and then the Kornblum reaction with DMSO to afford 1,6-oxyfluoroalkylated ketones **73**.

**Scheme 47:**
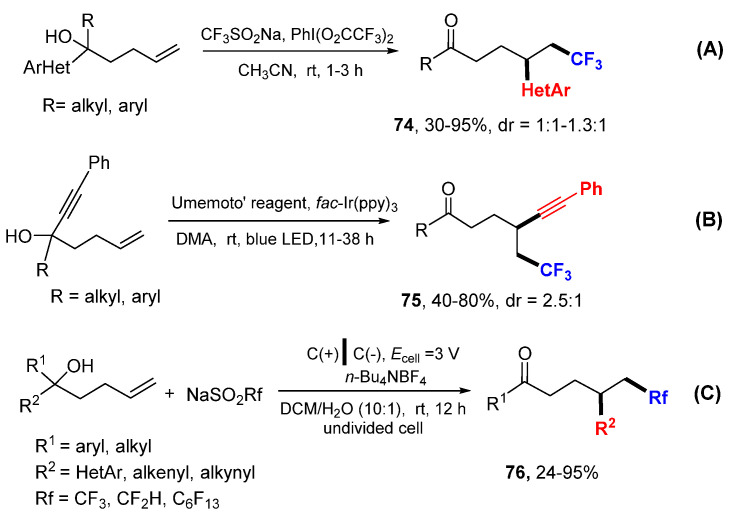
Fluorocarbon radical reactions involving distal group migration. (**A**) heteroaryl group migration reaction. (**B**) alkynyl group migration reaction. (**C**) electrochemical reaction conditions.

The Zhu group developed a method to generate CF_3_ radical from CF_3_SO_2_Na and PhI(O_2_CCF_3_)_2_ for difunctionalization of unactivated alkenes involving distal heteroaryl group migration to form products **74** (Scheme 47A) [79]. The Umemoto’s reagent is employed as CF_3_ radical source for trifluoromethylative alkynylation of unactivated alkenes to form products **75** through photocatalyzed reaction involving the alkynyl migration reaction (Scheme 47B) [80]. The Wang group reported an electrochemical reaction for fluoroalkylarylation of unactivated alkenes to form products **76** using R_f_SO_2_Na as fluorocarbon radical source (Scheme 47C) [81]. All these three reactions involve distal group migration and their mechanisms are similar to that presented in Scheme 14.

**Scheme 48:**
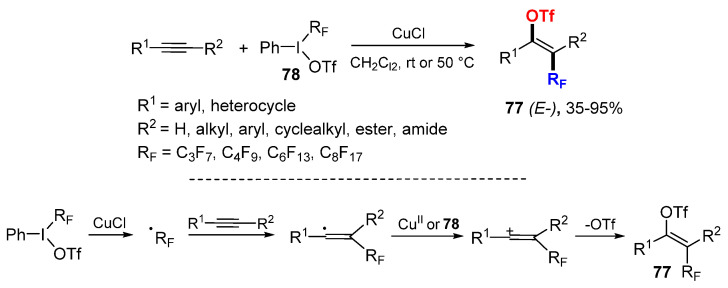
Perfluoroalkyltriflation of alkynes.

The Studer group reported a method for perfluoroalkyltriflation of alkynes using phenyl(perfluoroalkyl)iodonium triflates **78** in the presence of CuCl to afford selective *E-*difunctionalized alkene products **77** (Scheme 48) [82]. R_f_ radicals generated from **78** through reductive SET with CuCl add to alkynes to from vinyl radicals and then vinylic cations after oxidation. Trapping of cations with triflate anion affords the products **77**.

**Scheme 49:**
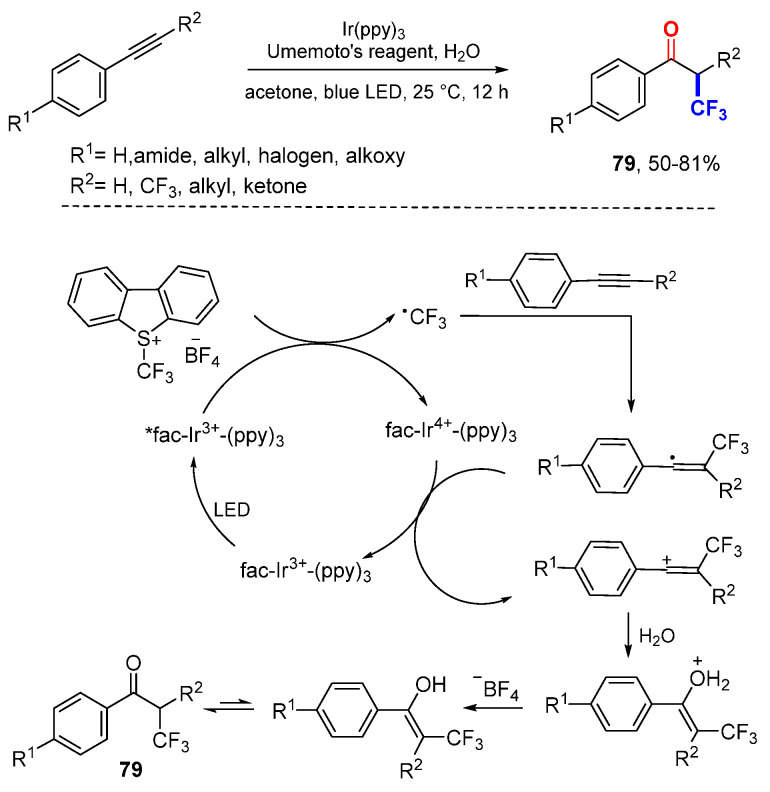
Ketotrifluoromethylation of alkynes. * excited catalyst.

Han and coworkers reported photoredox catalytic ketofluoromethylation of alkynes (Scheme 49) [83]. The CF_3_ radical generated from Umemoto’s reagent through reductive SET add to alkynes to produce vinyl radicals which are oxidized to vinyl cations by SET and then trapped by H_2_O to give products **79** after deprotonation with BF_4_^−^ and enol-keto tautomerization.

**Scheme 50:**
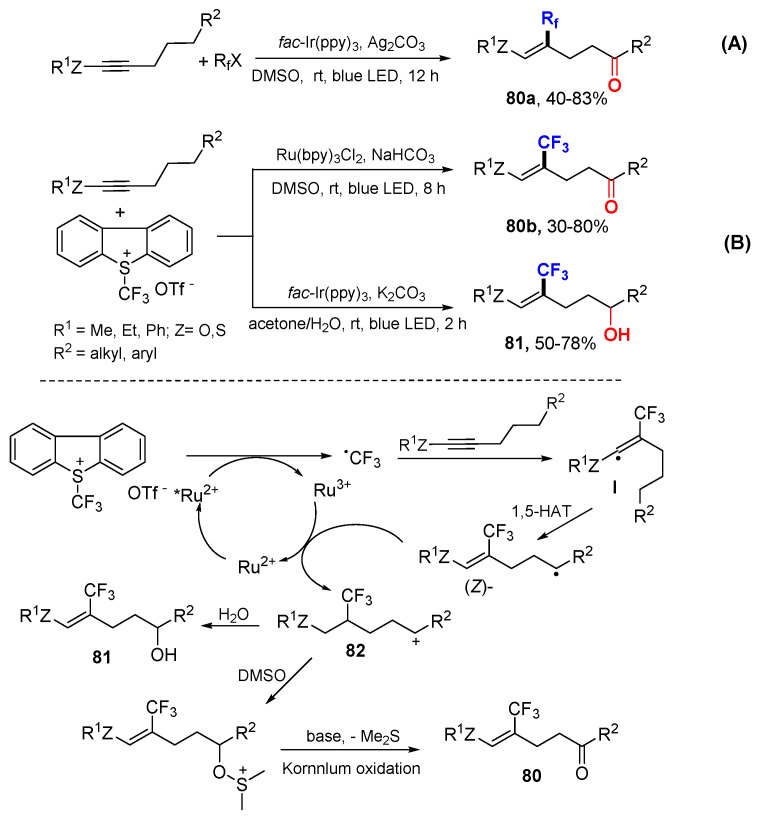
Photoredox-catalyzed remote oxyfluoroalkylation. (**A**) R_f_-involved reaction. (**B**) CF_3_-involved reaction. * excited catalyst.

The Zhu and Li group reported photoredox-catalyzed remote ketofluoroalkylation and hydroxytrifluoromethylation of alkynes using R_f_X or Umemoto’s reagent as fluoroalkyl source and DMSO or H_2_O as oxygen source for the synthesis of ε-oxygenated fluoroalkylated (*Z*)-alkenes **80** and **81** (Scheme 50) [84]. In the reaction with the Umemoto’s reagent, the CF_3_ radical generated through SET adds to alkynes to form vinyl radicals which then undergo 1,5-HAT followed by oxidative SET to form cationic intermediates **82** with a (*Z*)-alkenyl group. Nucleophilic attack of **82** by DMSO followed by the Kornblum oxidation produces trifluoromethyl (Z)-alkenyl ketones **80**. Alternatively, using H_2_O instead of DMSO as a nucleophile, trifluoromethylated (Z)-alkenols **81** are produced from the reaction.

## 8. Other Radical-Initiated Reactions

**Scheme 51:**
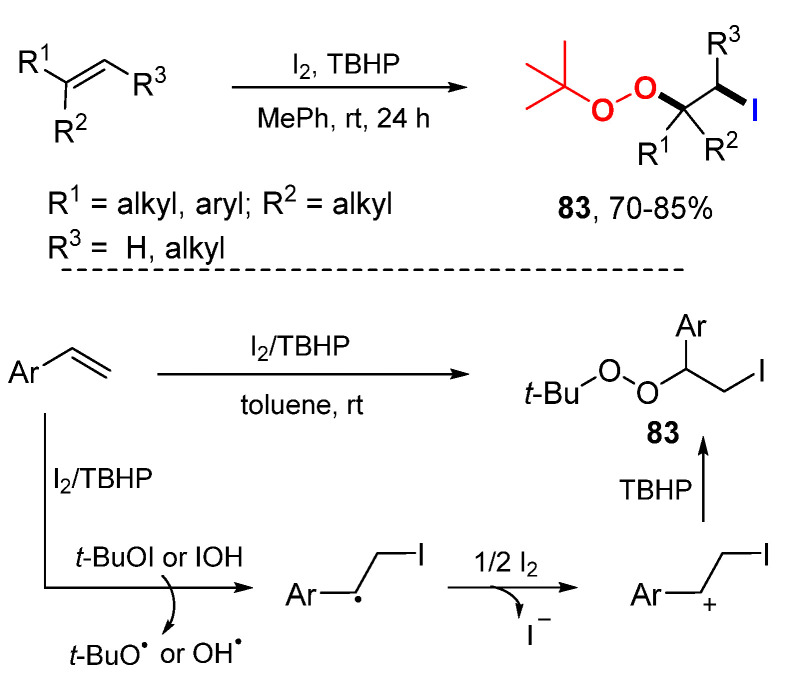
Iodoalkylperoxylation of alkenes.

Other than above mentioned difunctionalization reactions initiated with C-, N-, O-, P-, and fluorocarbon-radicals, we found a single example of iodo radical-initiated difunctionalization reported by the Zhu group (Scheme 51) [85]. The reaction of TBHP with I_2_ generates *t*-BuOI and HOI. Radical iodination of styrenes followed by oxidation to cations and nucleophilic attacking by TBHP give iodoalkylperoxylated products **83**.

## 9. Conclusions

This article summarizes the radical and nucleophilic difunctionalization reactions of alkenes and alkynes involving a cascade process of radical generation, radical addition, oxidation to cation, and trapping by a nucleophile. This one-pot reaction process demonstrates the step and pot economies as well as operational simplicity. A wide range C-, O-, N-, S-, P-centered and fluorocarbon radicals are used in combination with C-, N-, O-centered and halogenic nucleophiles for difunctionalizations to show substrate versatility.

Currently active research techniques such as photoredox catalysis and electrochemical reactions have been applied to the difunctionalization reactions. It is worth noting that in addition to radical and nucleophilic difunctionalization reactions presented in this paper, there are other radical-initiated difunctionalization reactions such as radical chain reactions, radical-radical coupling with or without metal catalysis. In some reaction processes, the radical difunctionalizations could have more than one pathways. The complex reaction processes have attracted the attention of synthetic chemists to conduct mechanism studies which could lead to the discovery of new reactions. We have no doubt that radical addition-initiated difunctionalization reactions for making molecules with potential biological, medical and functional material utilities will become important tools in organic synthesis.

## Data Availability

Data sharing not applicable.
